# Emergence of Novel Fluoroquinolone Resistance Mutations in *Mycoplasma bovis*, China, 2008–2023

**DOI:** 10.3201/eid3108.241137

**Published:** 2025-08

**Authors:** Shimei Lan, Shuang Liu, Wenjing Cui, Zhangcheng Li, Huafang Hao, Ahmed Adel Baz, Jinjia Liang, Xiangrui Jin, Xinmin Yan, Pengcheng Gao, Fuying Zheng, Shengli Chen, Yuefeng Chu

**Affiliations:** State Key Laboratory for Animal Disease Control and Prevention, College of Veterinary Medicine, Lanzhou University, Lanzhou Veterinary Research Institute, Chinese Academy of Agricultural Sciences, Lanzhou, China (S. Lan, S. Liu, W. Cui, Z. Li, H. Hao, A.A. Baz, J. Liang, X. Jin, X. Yan, P. Gao, F. Zheng, S. Chen, Y. Chu); Gansu Province Research Center for Basic Disciplines of Pathogen Biology, Lanzhou (S. Lan, S. Liu, Z. Li, H. Hao, A.A. Baz, J. Liang, X. Jin, X. Yan, P. Gao, F. Zheng, S. Chen, Y. Chu); Key Laboratory of Veterinary Etiological Biology, Key Laboratory of Ruminant Disease Prevention and Control (West), Ministry of Agricultural and Rural Affairs, Lanzhou (S. Lan, S. Liu, Z. Li, H. Hao, A.A. Baz, J. Liang, X. Jin, X. Yan, P. Gao, F. Zheng, S. Chen, Y. Chu)

**Keywords:** *Mycoplasma bovis*, multilocus sequence typing, MLST, sequence type, ST52, genetic evolutionary analysis, fluoroquinolone, fluoroquinolone resistance, antimicrobial resistance, bacteria, China

## Abstract

We investigated quinolone resistance in *Mycoplasma bovis* samples isolated in China during 2008–2023. Sequence type 52 was the dominant genotype; GyrA (S83F/Y) and ParC (S80R) protein double mutations caused high resistance to fluoroquinolones. Increased vigilance and surveillance of *M. bovis* infections in cattle will be needed to prevent disease.

Diseases in cattle caused by *Mycoplasma bovis* include bronchopneumonia, mastitis, and arthritis (*1*,*2*). *M. bovis* was first isolated in 1961 (*3*) and, over the past >6 decades, it has become widespread worldwide. Bovine mycoplasmosis caused by *M. bovis* is an emerging disease in China. Since the first isolation of *M. bovis* strains in China’s Hubei region in 2008, those strains have spread rapidly and extensively to most provinces in China (*4*–*6*). However, the epidemiologic features of *M. bovis* in China are unknown. Antimicrobial drugs are currently a critical means of controlling *M. bovis* infections (*7*,*8*). Fluoroquinolones have a substantial bactericidal effect against *Mycoplasma* spp.; however, their effectiveness has been gradually declining (*9*,*10*). Fluoroquinolone resistance in *Mycoplasma* spp. relies primarily on gene point mutations (*7*).

To elucidate molecular epidemiologic features of *M. bovis* in China, we performed a genetic evolutionary analysis of whole-genome sequences from 77 *M. bovis* isolates collected during 2008–2023 from 16 provinces in China; 34 isolates were identified in this study and 43 isolates were from GenBank (Appendix Table 1). We deposited sequence data for the *M. bovis* isolates from this study in the National Center for Biotechnology Information BioProject database (https://www.ncbi.nlm.nih.gov/bioproject; accession nos. PRJNA1124599–601). 

We explored the contribution of genetic factors to fluoroquinolone resistance. We confirmed that sequence type (ST) 52, the primary genotype responsible for the *M. bovis* infection outbreak in 2008, was the most prevalent genotype in China; however, the topologic structure of the phylogenetic tree classified the 77 isolates into 5 distinct clusters (I–V) (Figure 1). Five of those isolates represented new multilocus sequence typing (MLST) genotypes, which were primarily concentrated in cluster IV (which contained 4 MLST genotypes) (Figure 1; Appendix Figure 1), suggesting that isolates within cluster IV might have undergone rapid genetic changes. During the disease outbreak in 2008, ST53, ST56, and ST72 genotypes were also identified. Although those 3 genotypes were distributed sporadically, they have been isolated only in China and belong to the same clonal complex (CC) 52 as ST52 (Appendix Figure 1), exhibiting a high degree of genetic relatedness. That observation suggests that ST52 underwent genetic variation after spreading extensively in China. ST89 was isolated from cows with pneumonia and mastitis in China during 2018–2019 (Appendix Table 2); however, that genotype does not belong to CC52 (Appendix Figure 1). The isolation of only 3 ST89 strains suggested that strains with other genotypes might be infecting cattle in China.

**Figure 1:**
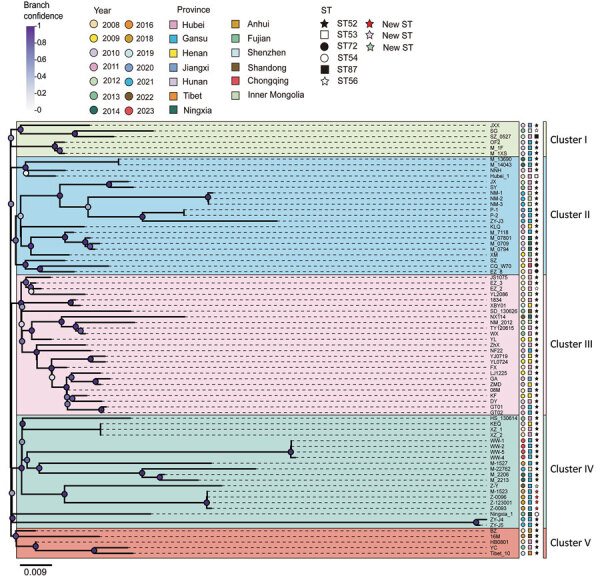
Phylogenetic analysis of *Mycoplasma bovis* in study of emergence of novel fluoroquinolone resistance mutations, China, 2008–2023. Maximum-likelihood tree shows 77 *M. bovis* isolates according to single-nucleotide polymorphisms identified by referencing the complete genome sequence of *M. bovis* strain HB0801. Name of isolate, year isolated, province, sequence type, and clustering are indicated. Scale bar indicates nucleotide substitutions per site.

We analyzed mutations within quinolone resistance–determining regions of the 77 isolated genomes from China. Mutations in those regions occurred primarily in *parC* and *gyrA* genes, leading to amino acid changes (Appendix Figure 2). Specifically, the GyrA protein contained S83Y and S83F mutations (Figure 2, panel A), and ParC contained S80R and D84G (Figure 2, panel B). The S80R mutation in ParC is uncommon in *M. bovis* and has not been reported in China. The binding energies of the GyrA S83F and S83Y and ParC S80R mutants with ciprofloxacin were higher than those for wild-type GyrA and ParC proteins. The mean + SD binding energy increased from −46.115 + 8.72 in wild-type GyrA to −10.242 + 2.892 in the GyrA S83Y mutant (Appendix Table 3). The ParC S80R mutant had considerably higher binding energy than wild-type ParC, increasing from −19.973 + 2.445 in wild-type protein to 26.861 + 5.14 in the mutant. Those mutations led to a decreased and unstable binding capacity of GyrA and ParC with ciprofloxacin.

**Figure 2:**
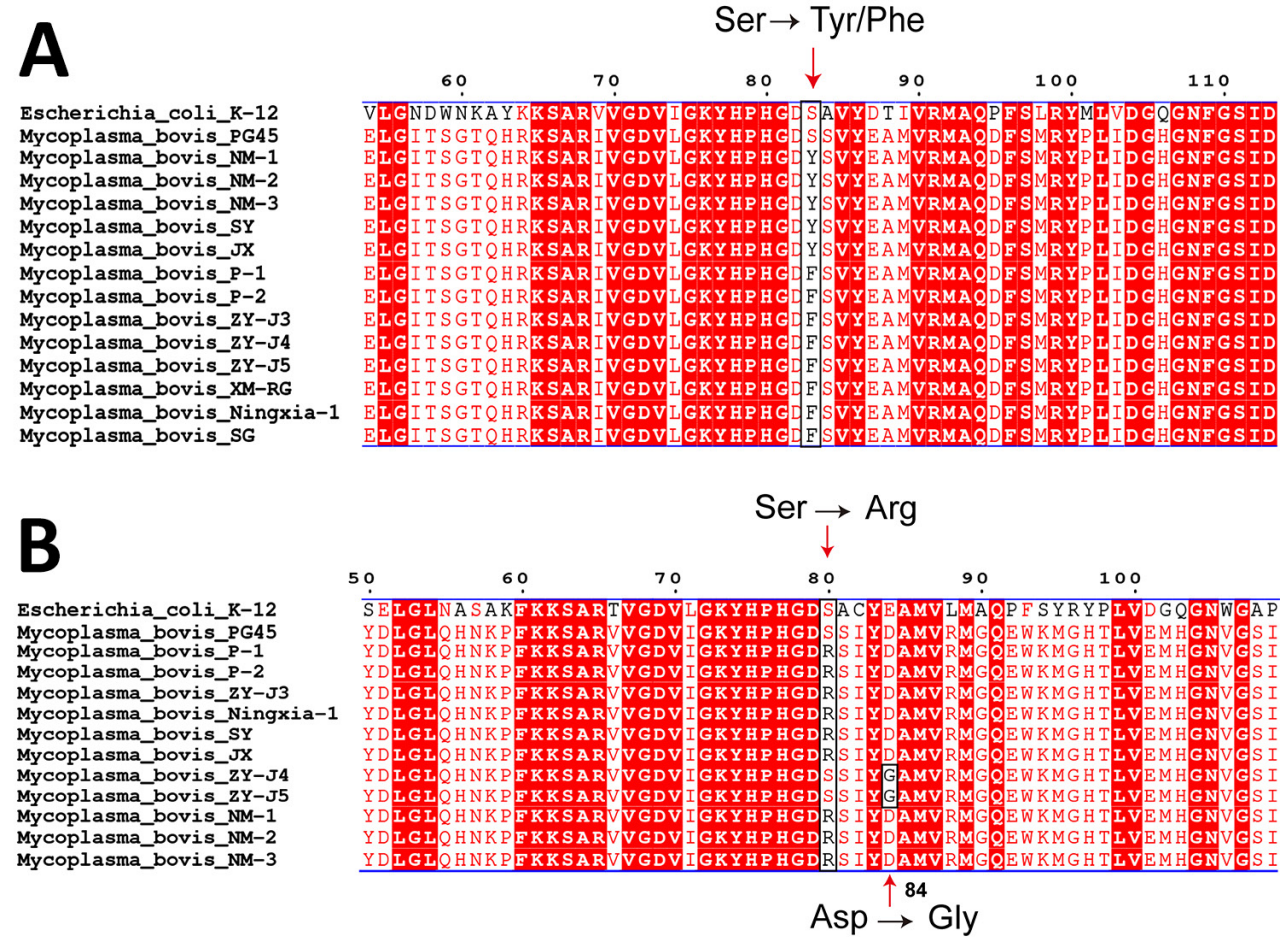
Amino acid sequence alignments of quinolone resistance-determining regions of *Mycoplasma bovis* isolates from China, 2008–2023. Multiple alignments of conserved GyrA (A) and ParC (B) protein sequences for *M. bovis* ParC protein–ciprofloxacin complex are shown. *Escherichia coli* K12 and *M. bovis* PG45 strains were used as controls. Red arrows and black rectangular borders indicate amino acid mutation sites.

We investigated the effect of mutations on fluoroquinolone susceptibility of *M. bovis*. Clinical isolates with the GyrA S83Y/F and ParC S80R double mutations exhibited lower susceptibility to fluoroquinolones than strains that had the GyrA S83F and ParC D84G double mutations (Appendix Table 4, Figure 3), suggesting that S83Y/F in GyrA combined with S80R in ParC conferred high resistance to fluoroquinolones; the S80R ParC mutation appeared to be the main reason for increased fluoroquinolone resistance. Molecular dynamic simulations revealed that residue S80 of *M. bovis* ParC interacts with enrofloxacin through van der Waals forces (Appendix Figure 4). Strains with GyrA and ParC mutations were mainly concentrated in cluster II (Figure 1), suggesting that cluster II strains are more prone to developing genetic features that confer resistance to fluoroquinolones.

In conclusion, we report that ST52 is the dominant *M. bovis* genotype circulating in China; however, ST52 strains gradually formed 2 subgroups with dominant genetic variation and fluoroquinolone resistance through widespread dissemination. The double mutation, S83F in GyrA and S80R in ParC, appears to be the current widespread mutation combination in China, and the emergence of high resistance to fluoroquinolones is driven by the ParC S80R mutation. Widespread resistance to fluoroquinolones poses a substantial challenge to the prevention and treatment of infections caused by *Mycoplasma* species; thus, increased vigilance and surveillance of *M. bovis* infections in cattle will be needed to prevent disease spread.

Appendix Additional information for emergence of novel fluoroquinolone resistance mutations in *Mycoplasma bovis*, China, 2008–2023.
