# Supplementary material for: Emergence of Novel Fluoroquinolone Resistance Mutations in Mycoplasma bovis, China, 2008–2023
Source: Emerg Infect Dis. 2025 Aug;31(8):1676–9. doi: 10.3201/eid3108.241137 (PMC12309742; doi:10.3201/eid3108.241137)
Supplement: Appendix — Additional information for emergence of novel fluoroquinolone resistance mutations in Mycoplasma bovis, China, 2008–2023. [file 24-1137-Techapp-s1.pdf]

Article DOI: https://doi.org/10.3201/eid3108.241137

*EID cannot ensure accessibility for supplementary materials supplied by authors. Readers who have difficulty accessing supplementary content should contact the authors for assistance.*

# Emergence of Novel Fluoroquinolone Resistance Mutations in *Mycoplasma bovis*, China, 2008–2023

## Appendix

### Additional Comments

Traceback analyses using the PubMLST database (https://pubmlst.org; 10 March 2024) revealed that sequence type (ST) 52 had been isolated from cattle in Australia and the United States as early as 2006, persisting throughout the subsequent ≈10-year period (*1*,*2*). A previous molecular epidemiologic study of *M. bovis* in Israel found that 87.5% of *M. bovis* isolates transmitted from Australia to Israel belonged to the ST52 genotype (*2*). Importantly, People's Republic of China and Israel have imported cattle from Australia over the past decade (*2*). Those findings suggest that ST52, which is disseminated widely in People's Republic of China, could share a common Australian origin with similar strains identified in Israel. This complexity underscores the global nature of the cattle trade and its implications for pathogen transmission.

Notably, studies have reported that a mutation from Asp to Asn at position 362 in the GyrB protein of *M. bovis* might increase resistance to fluoroquinolone antibiotics (*3*,*4*). However, we investigated 77 *M. bovis* strains prevalent in People's Republic of China and found that all exhibited the D362N mutation in GyrB (Appendix Figure 2, panel D). Despite this mutation, some isolates displayed extreme sensitivity to fluoroquinolone antibiotics. The *M. bovis* strains from People's Republic of China in our investigation belonged to the clonal complex (CC) 52, but corresponding mutations were not found in isolates from CC12 (Appendix

Figure 1, panel B; Appendix Figure 4). This observation suggests that the Asp→Asn mutation at position 362 in GyrB is not a necessary determinant for fluoroquinolone resistance in *M. bovis*, but may be associated with specific characteristics of certain genotypes.

## Additional Methods

### Data

Data on *M. bovis* isolates were obtained on 10 March 2024 from the PubMLST database (n = 1,315, comprising 824 characterized and 491 uncharacterized multilocus sequence typing (MLST) genotypes; Appendix Table 2). Then, *M. bovis* genomes from 16 provinces in People's Republic of China from 2008 to 2023 retrieved from GenBank (n = 43) and the whole-genome sequences of *M. bovis* strains from our study (n = 34) were used to explore the genetic evolutionary relationships of *M. bovis* in People's Republic of China (n = 77 in total) (Appendix Table 1).

### Phylogenetic Tree

Using the default settings of Snippy v4.6 (https://github.com/tseemann/snippy), comparative genomic analysis was conducted on 77 *M. bovis* genomes against the reference genome (*M. bovis* HB0801) employing BWA-MEM version 0.7.12 to map short reads. Snippy generated a "core complete alignment" file. Single-nucleotide polymorphisms (SNPs) were extracted and recombination sequences were removed by using Gubbins version 2.4.1 (*5*). Then, the SNP alignment file was used to construct a maximum-likelihood phylogenetic tree via FastTree v2.1.3 (www.microbesonline.org/fasttree), which was visualized and annotated subsequently by using tvBOT (*6*).

### Sequence Alignment and Visualization

Multiple sequence alignment of the amino acid sequences encoded by the *parC*, *gyrA*, and *gyrB* genes from *M. bovis* isolates was performed by using MAFFT 7.0 software (*7*). Subsequently, the alignment results were visualized by using the ESPript 3.0 server (*8*).

**Whole-Genome Sequencing**

The above mentioned 38 clinical strains isolated in People's Republic of China (n = 34), Hungary (n = 2), France (n = 1), and the United States (n = 1) and preserved in our laboratory underwent whole-genome sequencing (WGS). PacBio third-generation sequencing was used for 3 strains (08M, 07801, and N44), and Illumina (https://www.illumina.com) second-generation high-throughput sequencing was used for the remaining 35 strains.

**Illumina WGS**

For Illumina paired-end WGS, ≥3 μg genomic DNA per sample was used to construct sequencing libraries with ≈450-bp insert sizes, according to Illumina's protocol. The genomic DNA was fragmented to the desired size by using Covaris, followed by end repair using T4 DNA polymerase. Adenylation of the blunt ends facilitated adaptor ligation. Fragment selection was achieved through gel electrophoresis and subsequent PCR enrichment and indexing. Library quality was assessed before sequencing on an Illumina NovaSeq 6000 platform (150 bp × 2) by Shanghai Biozeron Biotechnology Co., Ltd (http://www.biozeron.com).

**PacBio WGS**

For PacBio WGS, PacBio Sequel IIe technology (Pacific Biosciences, https://www.pacb.com) was used; DNA was processed into SMRTbell libraries by using an Express Template Prep Kit 2.0 (Pacific Biosciences) per the manufacturer's instructions. The samples were combined into a single multiplexed library and underwent size selection via Sage Sciences' BluePippin by following the 0.75% Dye-Free (DF) Marker S1 High-Pass 6–10 kb v3 protocol with a cutoff of 8000 bp. Thereafter, the SMRTbell library was prepared for sequencing by annealing and binding according to the SMRT Link Set Up guidelines on the Sequel IIe system.

**Genome Assembly**

Raw paired-end reads were trimmed and quality controlled using Trimmomatic, producing clean data for further analysis. PacBio reads were converted to FASTQ format via

Samtools. Illumina datasets were employed to assess genome complexity and to correct the PacBio long reads. Genome assembly was conducted with Unicycler v0.4.8 using default settings, yielding optimal assembly outcomes. GC content, depth, and genome size were determined using custom Perl scripts to detect potential contamination. Each assembled strain genome was circularized using Circlator version 1.5.5.

**Molecular Dynamic Simulations**

Each protein structure (ParC and GyrA) was converted into a pdbqt file using AutoDockTools v4.2.6 within MGLTools software (*9*). Each small-molecule ligand (enrofloxacin, danofloxacin, and ciprofloxacin) was also processed into pdbqt files using AutoDockTools. A docking box was constructed to encompass the entire protein. Each small-molecule ligand was docked with the protein using Autodock Vina, followed by an analysis of the docking results to evaluate their conformations.

Subsequently, each protein and small-molecule ligand from the docking results were separated. The Antechamber tool within AmberTools version 23 (*10*) was used to generate a force field file for the small-molecule ligand, which was then converted into a Gromacs force field file using the ACPYPE software tool (*11*). The small-molecule ligand was modeled using the GAFF force field, whereas the protein was simulated with the AMBER14SB force field and TIP3P water model. Files for the protein and small-molecule ligand were merged to construct a simulation system for the complex. Molecular dynamic (MD) simulations were conducted using the Gromacs2022 program, under constant temperature and pressure conditions with periodic boundary conditions. Throughout the MD simulation, all interactions involving hydrogen bonds were constrained using the LINear Constraint Solver algorithm, with an integration step size of 2 fs. The electrostatic interactions were calculated using the particle-mesh Ewald method with a cutoff of 1.2 nm. Nonbonded interactions had a cutoff of 10 Å, with an update every 10 steps. The V-rescale thermostat method was employed to maintain the simulation temperature at 298 K, while the Berendsen barostat method was used to maintain the pressure at 1 bar. At 298 K, equilibration simulations of both NVT (constant number of particles, volume, and temperature)

and NPT (constant number of particles, pressure, and temperature) were performed for 100 ps, followed by a 100-ns MD simulation of the complex; conformations were saved every 10 ps. Upon completion of the simulation, the trajectory was analyzed, and the MM-PBSA binding free energy of the complex was calculated using the g_mmpbsa program.

#### MIC Assays

Pleuropneumonia-like organisms (PPLO) broth was supplemented with 2 g/L sodium pyruvate, 2 g/L glucose, 10% yeast extract, and 10% horse serum to create a culture medium (pH 7.6–7.8) for *Mycoplasma* species. For each MIC assay, antibiotic dilutions were prepared fresh according to instructions. There were 2-fold serial dilutions (ranging from 0.0625 to 128 µg/mL) of fluoroquinolones comprising enrofloxacin (catalog number E302224–1g; Solarbio, https://www.solarbio.com), danofloxacin (D132445–100mg; Solarbio), and ciprofloxacin (C304792–25 g; Solarbio). MIC assays were conducted in accordance with the recommendations of Hannan and colleagues (*12*) and Gütgemann and collaborators (*13*) with slight modifications using an inoculation concentration of $10^3$ colony forming units/mL for each tested strain. Three replicates of each of the clinical isolates and reference strain (*M. bovis* PG45) were tested in 96-well microtiter plates. In addition, wells in the 96-well microtiter plates were used for growth control (culture medium containing inoculums of *Mycoplasma* species but no antibiotics), sterility control (culture medium containing neither antibiotics nor inoculums of *Mycoplasma* species), and drug control (culture medium containing only the lowest antibiotic dilution).

#### Data Availability

Publicly available sequence data were downloaded from PubMLST (https://pubmlst.org/organisms/mycoplasma-bovis) and NCBI (https://www.ncbi.nlm.nih.gov/datasets/genome/?taxon=28903).

**References**

1. Register KB, Lysnyansky I, Jelinski MD, Boatwright WD, Waldner M, Bayles DO, et al. Comparison of two multilocus sequence typing schemes for *Mycoplasma bovis* and revision of the PubMLST reference method. J Clin Microbiol. 2020;58:e00283-20. PubMed https://doi.org/10.1128/JCM.00283-20

2. Yair Y, Borovok I, Mikula I, Falk R, Fox LK, Gophna U, et al. Genomics-based epidemiology of bovine *Mycoplasma bovis* strains in Israel. BMC Genomics. 2020;21:70. PubMed https://doi.org/10.1186/s12864-020-6460-0

3. Khalil D, Becker CAM, Tardy F. Alterations in the quinolone resistance-determining regions and fluoroquinolone resistance in clinical isolates and laboratory-derived mutants of *Mycoplasma bovis*: not all genotypes may be equal. Appl Environ Microbiol. 2015;82:1060–8. PubMed https://doi.org/10.1128/AEM.03280-15

4. Gautier-Bouchardon AV. Antimicrobial resistance in *Mycoplasma* spp. Microbiol Spectr. 2018;6:10.1128/microbiolspec.arba-0030-2018. PubMed https://doi.org/10.1128/microbiolspec.ARBA-0030-2018

5. Croucher NJ, Page AJ, Connor TR, Delaney AJ, Keane JA, Bentley SD, et al. Rapid phylogenetic analysis of large samples of recombinant bacterial whole genome sequences using Gubbins. Nucleic Acids Res. 2015;43:e15. PubMed https://doi.org/10.1093/nar/gku1196

6. Xie J, Chen Y, Cai G, Cai R, Hu Z, Wang H. Tree Visualization By One Table (tvBOT): a web application for visualizing, modifying and annotating phylogenetic trees. Nucleic Acids Res. 2023;51:W587–92. PubMed https://doi.org/10.1093/nar/gkad359

7. Katoh K, Standley DM. MAFFT multiple sequence alignment software version 7: improvements in performance and usability. Mol Biol Evol. 2013;30:772–80. PubMed https://doi.org/10.1093/molbev/mst010

8. Robert X, Gouet P. Deciphering key features in protein structures with the new ENDscript server. Nucleic Acids Res. 2014;42:W320–4. PubMed https://doi.org/10.1093/nar/gku316

9. Arcon JP, Modenutti CP, Avendaño D, Lopez ED, Defelipe LA, Ambrosio FA, et al. AutoDock Bias: improving binding mode prediction and virtual screening using known protein-ligand interactions. Bioinformatics. 2019;35:3836–8. PubMed https://doi.org/10.1093/bioinformatics/btz152

10. Case DA, Aktulga HM, Belfon K, Cerutti DS, Cisneros GA, Cruzeiro VWD, et al. AmberTools. J Chem Inf Model. 2023;63:6183–91. PubMed https://doi.org/10.1021/acs.jcim.3c01153

11. Kagami L, Wilter A, Diaz A, Vranken W. The ACPYPE web server for small-molecule MD topology generation. Bioinformatics. 2023;39:btad350. PubMed https://doi.org/10.1093/bioinformatics/btad350

12. Hannan PC. Guidelines and recommendations for antimicrobial minimum inhibitory concentration (MIC) testing against veterinary *Mycoplasma* species. International Research Programme on Comparative Mycoplasmology. Vet Res. 2000;31:373–95. PubMed https://doi.org/10.1051/vetres:2000100

13. Gütgemann F, Müller A, Churin Y, Kumm F, Braun AS, Yue M, et al. Toward a method for harmonized susceptibility testing of *Mycoplasma bovis* by broth microdilution. J Clin Microbiol. 2023;61:e0190522. PubMed https://doi.org/10.1128/jcm.01905-22

**Appendix Table 1.** Whole-genome sequencing data for *Mycoplasma bovis* strains from this study and GenBank

| Strain | Country | Region | GenBank accession no.* |
|---|---|---|---|
| *M. bovis* GT01 | China | Asia | This study |
| *M. bovis* GT02 | China | Asia | This study |
| *M. bovis* 1523 | China | Asia | This study |
| *M. bovis* 1527 | China | Asia | This study |
| *M. bovis* 22762 | China | Asia | This study |
| *M. bovis* 0709 | China | Asia | This study |
| *M. bovis* 07801 | China | Asia | This study |
| *M. bovis* 0794 | China | Asia | This study |
| *M. bovis* 13690 | China | Asia | This study |

| Strain | Country | Region | GenBank accession no.* |
|---|---|---|---|
| *M. bovis* 14043 | China | Asia | This study |
| *M. bovis* 1F | China | Asia | This study |
| *M. bovis* 1XS | China | Asia | This study |
| *M. bovis* 2206 | China | Asia | This study |
| M. bovis 2213 | China | Asia | This study |
| *M. bovis* 7118 | China | Asia | This study |
| *M. bovis* NF22 | China | Asia | This study |
| *M. bovis* NM-1 | China | Asia | This study |
| *M. bovis* NM-2 | China | Asia | This study |
| *M. bovis* NM-3 | China | Asia | This study |
| *M. bovis* OF2 | China | Asia | This study |
| *M. bovis* P-1 | China | Asia | This study |
| *M. bovis* P-2 | China | Asia | This study |
| *M. bovis* WW-1 | China | Asia | This study |
| *M. bovis* WW-2 | China | Asia | This study |
| *M. bovis* WW-4 | China | Asia | This study |
| *M. bovis* WW-5 | China | Asia | This study |
| *M. bovis* Z-0093 | China | Asia | This study |
| *M. bovis* Z-0096 | China | Asia | This study |
| *M. bovis* Z-123001 | China | Asia | This study |
| *M. bovis* Z-Y | China | Asia | This study |
| *M. bovis* ZY-J3 | China | Asia | This study |
| *M. bovis* ZY-J4 | China | Asia | This study |
| *M. bovis* ZY-J5 | China | Asia | This study |
| *M. bovis* 08M | China | Asia | GCF_002009275.1_ASM200927v1_genomic (our lab) |
| *M. bovis* NX114 | China | Asia | GCF_032463445.1_ASM3246344v1_genomic |
| *M. bovis* Tibet-10 | China | Asia | GCF_014854615.1_ASM1485461v1_genomic |
| *M. bovis* XBY01 | China | Asia | GCF_009650115.1_ASM965011v1_genomic |
| *M. bovis* FX | China | Asia | GCF_006659345.1_ASM665934v1_genomic |
| *M. bovis* EZ-2 | China | Asia | GCF_006659305.1_ASM665930v1_genomic |
| *M. bovis* ZMD | China | Asia | GCF_006659275.1_ASM665927v1_genomic |
| *M. bovis* KEQ | China | Asia | GCF_006659265.1_ASM665926v1_genomic |
| *M. bovis* XM-RG | China | Asia | GCF_006659245.1_ASM665924v1_genomic |
| *M. bovis* GA | China | Asia | GCF_006659195.1_ASM665919v1_genomic |
| *M. bovis* BZ | China | Asia | GCF_006659175.1_ASM665917v1_genomic |
| *M. bovis* SG | China | Asia | GCF_006659165.1_ASM665916v1_genomic |
| *M. bovis* XZ-1 | China | Asia | GCF_006659155.1_ASM665915v1_genomic |
| *M. bovis* SD-130626 | China | Asia | GCF_006659145.1_ASM665914v1_genomic |
| *M. bovis* YLrengong | China | Asia | GCF_006659125.1_ASM665912v1_genomic |
| *M. bovis* YC | China | Asia | GCF_006659075.1_ASM665907v1_genomic |

| Strain | Country | Region | GenBank accession no.* |
|---|---|---|---|
| *M. bovis* WX | China | Asia | GCF_006659065.1_ASM665906v1_genomic |
| *M. bovis* JX | China | Asia | GCF_006659055.1_ASM665905v1_genomic |
| *M. bovis* KLQ | China | Asia | GCF_006659045.1_ASM665904v1_genomic |
| *M. bovis* DY | China | Asia | GCF_006659025.1_ASM665902v1_genomic |
| *M. bovis* EZ-8 | China | Asia | GCF_006658975.1_ASM665897v1_genomic |
| *M. bovis* XM | China | Asia | GCF_006658965.1_ASM665896v1_genomic |
| *M. bovis* SZ | China | Asia | GCF_006658955.1_ASM665895v1_genomic |
| *M. bovis* ZhX | China | Asia | GCF_006658945.1_ASM665894v1_genomic |
| *M. bovis* YJ0719 | China | Asia | GCF_006658905.1_ASM665890v1_genomic |
| *M. bovis* YL0724 | China | Asia | GCF_006658885.1_ASM665888v1_genomic |
| *M. bovis* NNH | China | Asia | GCF_006658865.1_ASM665886v1_genomic |
| *M. bovis* F150tu | China | Asia | GCF_006658855.1_ASM665885v1_genomic |
| *M. bovis* JS1075 | China | Asia | GCF_006658845.1_ASM665884v1_genomic |
| *M. bovis* 1834 | China | Asia | GCF_006658825.1_ASM665882v1_genomic |
| *M. bovis* TY120615 | China | Asia | GCF_006658795.1_ASM665879v1_genomic |
| *M. bovis* DYrengong | China | Asia | GCF_006658785.1_ASM665878v1_genomic |
| *M. bovis* F150niu | China | Asia | GCF_006658745.1_ASM665874v1_genomic |
| *M. bovis* SY | China | Asia | GCF_006658735.1_ASM665873v1_genomic |
| *M. bovis* LJ1225 | China | Asia | GCF_006658725.1_ASM665872v1_genomic |
| *M. bovis* KF | China | Asia | GCF_006658695.1_ASM665869v1_genomic |
| *M. bovis* JXXY | China | Asia | GCF_006658685.1_ASM665868v1_genomic |
| *M. bovis* YL2086 | China | Asia | GCF_006658645.1_ASM665864v1_genomic |
| *M. bovis* YL | China | Asia | GCF_006658585.1_ASM665858v1_genomic |
| *M. bovis* SZ-0527 | China | Asia | GCF_006542475.1_ASM654247v1_genomic |
| *M. bovis* EZ-3 | China | Asia | GCF_006542465.1_ASM654246v1_genomic |
| *M. bovis* XZ-2 | China | Asia | GCF_006542455.1_ASM654245v1_genomic |
| *M. bovis* 16M | China | Asia | GCF_004792535.1_ASM479253v1_genomic |
| *M. bovis* HS-130614 | China | Asia | GCF_004751945.1_ASM475194v1_genomic |
| *M. bovis* Ningxia-1 | China | Asia | GCF_002749575.1_ASM274957v1_genomic |
| *M. bovis* NM 2012 | China | Asia | GCF_001043135.1_ASM104313v1_genomic |
| *M. bovis* CQ-W70 | China | Asia | GCF_000696015.1_ASM69601v1_genomic |
| *M. bovis* HB0801 | China | Asia | GCF_000270525.1_ASM27052v1_genomic |
| *M. bovis* Hubei-1 | China | Asia | GCF_000219375.1_ASM21937v1_genomic |
| *M. bovis* 970139 | France | Europe | In this study |
| *M. bovis* N43 | Hungary | Europe | In this study |
| *M. bovis* N44 | Hungary | Europe | In this study |
| *M. bovis* Madison | USA | North America | In this study |
| *M. bovis* PG45 | USA | North America | GCF_000183385.1_ASM18338v1_genomic |

*Sequences from this study were deposited in the NCBI BioProject database (https://www.ncbi.nlm.nih.gov/bioproject; accession nos. PRJNA1124599–601).

**Appendix Table 2.** *Mycoplasma bovis* isolates from the PubMLST global database*

| Isolate name | Host | Site | Country | Continent | Year | ST |
|---|---|---|---|---|---|---|
| HB0801 | bovid | lung | China | Asia | 2008 | 52 |
| 63307 | bovid | lung | Australia | Oceania | 2010 | 52 |
| 86812 | bovid | milk | Israel | Asia | 2010 | 52 |
| HAZ1734 | bovid | nasal cavity | Japan | Asia | 2014 | 52 |
| NM2012 | bovid | joint | China | Asia | 2012 | 52 |
| 08M | bovid | lung | China | Asia | 2008 | 52 |
| MJ24 | bovid | lung | Canada | North America | 2015 | 52 |
| MJ30 | bovid | lung | Canada | North America | 2007 | 52 |
| MJ63 | bovid | lung | Canada | North America | 2008 | 52 |
| MJ64 | bovid | joint | Canada | North America | 2007 | 52 |
| MJ94 | bovid | ear | Canada | North America | 2015 | 52 |
| MJ246 | bovid | joint | Canada | North America | 2016 | 52 |
| 6099 | bovid | milk | Israel | Asia | 2007 | 52 |
| 347 | bovid | milk | Israel | Asia | 2008 | 52 |
| 432 | bovid | milk | Israel | Asia | 2008 | 52 |
| 928 | bovid | milk | Israel | Asia | 2008 | 52 |
| 758 | bovid | milk | Israel | Asia | 2008 | 52 |
| 2621 | bovid | milk | Israel | Asia | 2008 | 52 |
| 2622 | bovid | milk | Israel | Asia | 2008 | 52 |
| 5428 | bovid | milk | Israel | Asia | 2008 | 52 |
| 2715 | bovid | milk | Israel | Asia | 2008 | 52 |
| 110 | bovid | milk | Israel | Asia | 2008 | 52 |
| 1662 | bovid | milk | Israel | Asia | 2008 | 52 |
| 991–2 | bovid | milk | Israel | Asia | 2008 | 52 |
| 889 | bovid | milk | Israel | Asia | 2008 | 52 |
| 10–155 | bovid | milk | Israel | Asia | 2008 | 52 |
| 783 | bovid | milk | Israel | Asia | 2009 | 52 |
| 26443 | bovid | milk | Israel | Asia | 2009 | 52 |
| 701 | bovid | milk | Israel | Asia | 2009 | 52 |
| 299 | bovid | milk | Israel | Asia | 2010 | 52 |
| 883 | bovid | milk | Israel | Asia | 2010 | 52 |
| 65714 | bovid | milk | Israel | Asia | 2011 | 52 |
| 111449 | bovid | milk | Israel | Asia | 2011 | 52 |
| 108432 | bovid | milk | Israel | Asia | 2011 | 52 |
| 127377 | bovid | milk | Israel | Asia | 2012 | 52 |
| 126814 | bovid | milk | Israel | Asia | 2012 | 52 |
| 129771 | bovid | milk | Israel | Asia | 2012 | 52 |
| 147529 | bovid | milk | Israel | Asia | 2012 | 52 |
| 139667 | bovid | milk | Israel | Asia | 2013 | 52 |

| Isolate name | Host | Site | Country | Continent | Year | ST |
|---|---|---|---|---|---|---|
| 170217 | bovid | milk | Israel | Asia | 2013 | 52 |
| 170228 | bovid | milk | Israel | Asia | 2013 | 52 |
| 178843 | bovid | milk | Israel | Asia | 2013 | 52 |
| XBY01 | bovid | lung | China | Asia | 2019 | 52 |
| 209716 | bovid | milk | Israel | Asia | 2014 | 52 |
| 220642 | bovid | milk | Israel | Asia | 2015 | 52 |
| 222991 | bovid | milk | Israel | Asia | 2015 | 52 |
| 227457 | bovid | milk | Israel | Asia | 2015 | 52 |
| 227456 | bovid | milk | Israel | Asia | 2015 | 52 |
| 227455 | bovid | milk | Israel | Asia | 2015 | 52 |
| 227465 | bovid | milk | Israel | Asia | 2015 | 52 |
| 228404 | bovid | milk | Israel | Asia | 2015 | 52 |
| 254410 | bovid | milk | Israel | Asia | 2016 | 52 |
| 280413 | bovid | milk | Israel | Asia | 2016 | 52 |
| 290360 | bovid | milk | Israel | Asia | 2017 | 52 |
| 161801 | bovid | milk | Russia | Asia | 2013 | 52 |
| 2A | bovid | pharynx | Australia | Oceania | 2006 | 52 |
| H | bovid | joint | Australia | Oceania | 2006 | 52 |
| 2D | bovid | pharynx | Australia | Oceania | 2006 | 52 |
| 1254 | bovid | lung | Australia | Oceania | 2006 | 52 |
| 261552 | bovid | lung | Australia | Oceania | 2016 | 52 |
| 41569 | bovid | lung | Australia | Oceania | 2009 | 52 |
| 2583 | bovid | larynx | Australia | Oceania | 2009 | 52 |
| 261553–9503 | bovid | joint | Australia | Oceania | 2016 | 52 |
| 261553–9655 | bovid | joint | Australia | Oceania | 2016 | 52 |
| 261552–3688 | bovid | lung | Australia | Oceania | 2016 | 52 |
| 261552–2741 | bovid | lung | Australia | Oceania | 2016 | 52 |
| 3893 | bovid | lung | Hungary | Europe | 2007 | 52 |
| 5180 | bovid | lung | Israel | Asia | 2006 | 52 |
| 5028 | bovid | lung | Israel | Asia | 2008 | 52 |
| 18525 | bovid | lung | Israel | Asia | 2008 | 52 |
| 70262–1 | bovid | vulva | Israel | Asia | 2010 | 52 |
| 72211 | bovid | vulva | Israel | Asia | 2010 | 52 |
| 219363 | bovid | lung | Israel | Asia | 2015 | 52 |
| 268681 | bovid | eye | Israel | Asia | 2016 | 52 |
| 270940 | bovid | lung | Israel | Asia | 2016 | 52 |
| 287942 | bovid | lung | Israel | Asia | 2017 | 52 |
| 236–22 | bovid | milk | USA | North America | 2008 | 52 |
| 236–27 | bovid | milk | USA | North America | 2008 | 52 |
| 236–28 | bovid | milk | USA | North America | 2008 | 52 |

| Isolate name | Host | Site | Country | Continent | Year | ST |
|---|---|---|---|---|---|---|
| 236–48 | bovid | milk | USA | North America | 2007 | 52 |
| 237–4 | bovid | milk | USA | North America | 2006 | 52 |
| JS1075-NHD0955 | bovid | lung | China | Asia | 2008 | 52 |
| SZ-NHD0960 | bovid | lung | China | Asia | 2008 | 52 |
| 1834-NHD0953 | bovid | lung | China | Asia | 2008 | 52 |
| EZ-3-NHD0947 | bovid | lung | China | Asia | 2008 | 52 |
| XZ-1-NHD0981 | bovid | lung | China | Asia | 2008 | 52 |
| XZ-2-NHD0946 | bovid | lung | China | Asia | 2008 | 52 |
| FX-NHD0970 | bovid | lung | China | Asia | 2008 | 52 |
| NNH-NHD0956 | bovid | larynx | China | Asia | 2010 | 52 |
| ZhX | bovid | lung | China | Asia | 2010 | 52 |
| DY-NHD0963 | bovid | lung | China | Asia | 2010 | 52 |
| TY-120615-NHD0952 | bovid | lung | China | Asia | 2012 | 52 |
| JX-NHD0966 | bovid | lung | China | Asia | 2012 | 52 |
| F150tu-NHD0954 | bovid | lung | China | Asia | 2012 | 52 |
| F150niu-NHD0949 | bovid | lung | China | Asia | 2012 | 52 |
| Dyrengong-NHD0951 | bovid | lung | China | Asia | 2012 | 52 |
| HB0801-rengong NHD0989 | bovid | lung | China | Asia | 2012 | 52 |
| SY-NHD0950 | bovid | lung | China | Asia | 2013 | 52 |
| WX-NHD0964 | bovid | joint | China | Asia | 2013 | 52 |
| YC-NHD0967 | bovid | milk | China | Asia | 2013 | 52 |
| BZ-NHD0982 | bovid | lung | China | Asia | 2008 | 52 |
| XM | bovid | lung | China | Asia | 2009 | 52 |
| XMrengong-NHD0985 | bovid | lung | China | Asia | 2012 | 52 |
| LJ1225-NHD0945 | bovid | lung | China | Asia | 2009 | 52 |
| YL-NHD0941 | bovid | lung | China | Asia | 2009 | 52 |
| KF | bovid | lung | China | Asia | 2009 | 52 |
| YL0724-NHD0957 | bovid | lung | China | Asia | 2009 | 52 |
| YJ0719-NHD0958 | bovid | lung | China | Asia | 2012 | 52 |
| KEQ-NHD0988 | bovid | lung | China | Asia | 2010 | 52 |
| KLQ | bovid | lung | China | Asia | 2010 | 52 |
| ZMD | bovid | lung | China | Asia | 2011 | 52 |
| YLrengong-NHD0968 | bovid | lung | China | Asia | 2012 | 52 |
| YL2086 | bovid | lung | China | Asia | 2012 | 52 |
| JXXY | bovid | lung | China | Asia | 2012 | 52 |
| GA-NHD0984 | bovid | lung | China | Asia | 2012 | 52 |
| SG-NHD0983 | bovid | lung | China | Asia | 2013 | 52 |
| SD-130626-NHD0969 | bovid | lung | China | Asia | 2013 | 52 |
| 16M | bovid | lung | China | Asia | 2016 | 52 |
| HS-130614 | bovid | unknown | China | Asia | 2013 | 52 |

| Isolate name | Host | Site | Country | Continent | Year | ST |
|---|---|---|---|---|---|---|
| JZBTM | bovid | milk | China | Asia | 2019 | 52 |
| HBXTBTM | bovid | milk | China | Asia | 2019 | 52 |
| SD1901 | bovid | milk | China | Asia | 2019 | 52 |
| MJ255 | bovid | lung | Canada | North America | 2017 | 52 |
| MJ256 | bovid | joint | Canada | North America | 2017 | 52 |
| MJ257 | bovid | lung | Canada | North America | 2017 | 52 |
| MJ270 | bovid | joint | Canada | North America | 2017 | 52 |
| MJ279 | bovid | lung | Canada | North America | 2017 | 52 |
| MPLM0631 | bovid | joint | Canada | North America | 2007 | 52 |
| MB651 | calf | lung | Turkey | Asia | 2021 | 52 |
| Hubei-1 | bovid | lung | China | Asia | 2008 | 53 |
| Ningxia-1 | bovid | lung | China | Asia | 2013 | 54 |
| EZ-2 | bovid | unknown | China | Asia | 2008 | 56 |
| CQ-W70 | bovid | lung | China | Asia | 2009 | 72 |
| EZ-8-NHD0962 | bovid | lung | China | Asia | 2008 | 72 |
| SZ-0527 | bovid | unknown | China | Asia | 2012 | 87 |
| NMH7 | bovid | milk | China | Asia | 2018 | 89 |
| NMH10 | bovid | milk | China | Asia | 2018 | 89 |
| HBCB01 | bovid | lung | China | Asia | 2019 | 89 |
| ShaanxiBTM01 | bovid | milk | China | Asia | 2018 | 90 |
| HBBDBTM | bovid | milk | China | Asia | 2018 | 90 |
| HBLFBTM01 | bovid | milk | China | Asia | 2018 | 90 |

*PubMLST database (https://pubmlst.org). ST, sequence type.

**Appendix Table 3.** Calculation of the binding free energies of wild-type and mutant *Mycoplasma bovis* GyrA and ParC proteins with ciprofloxacin*

| | Binding energy, KJ/mol (SD) | | | | | | |
|---|---|---|---|---|---|---|---|
| Complex† | $\Delta E_{vdw}$ | $\Delta E_{ele}$ | $\Delta E_{pol}$ | $\Delta E_{nonpol}$ | $\Delta E_{MMPBSA}$‡ | $-T\Delta S$ | $\Delta G_{bind}$§ |
| GyrA | | | | | | | |
| Wild type | –176.75 (6.949) | –49.182 13.173) | 179.361 (19.121) | –22.275 (0.057) | –68.845 (6.711) | 22.730 (2.408) | –46.115 (8.72) |
| S150F | –116.887 (1.918) | –7.279 (2.208) | 77.115 (3.760) | –16.375 (0.276) | –63.426 (0.139) | 19.997 (0.599) | –43.429 (0.500) |
| S150Y | –128.426 (2.159) | –28.583 (1.907) | 136.495 (6.246) | –17.798 (0.248) | –38.313 (5.766) | 28.071 (3.504) | –10.242 (2.892) |
| ParC | | | | | | | |
| Wild type | –124.659 (4.152) | –22.821 (7.459) | 102.890 (13.640) | –16.802 (0.471) | –61.392 (4.670) | 41.418 (3.982) | –19.973 (2.445) |
| S91R | –114.985 (5.927) | –61.062 (4.335) | 179.926 (9.898) | –17.683 (0.723) | –13.804 (0.563) | 40.665 (4.608) | 26.861 (5.140) |

*$\Delta E_{ele}$, electrostatic energy; $\Delta E_{MMPBSA}$, binding energy; $\Delta E_{nonpol}$, nonpolar solvation energy; $\Delta E_{pol}$, polar solvation energy; $\Delta E_{vdw}$, van der Walls energy; $-T\Delta S$, entropic contribution.

†*Escherichia coli* K12 strain GyrA mutation S150F corresponds to the S83F mutation and *E.coli* S150Y corresponds to the S83Y mutation in GyrA of *M. bovis* isolates. *Escherichia coli* K12 strain S91R mutation in ParC corresponds to the S80R mutation site in ParC of *M. bovis* isolates.

‡$\Delta E_{MMPBSA} = \Delta E_{ele} + \Delta E_{vdw} + \Delta E_{pol} + \Delta E_{nonpol}$.

§$\Delta G_{bind} = \Delta E_{MMPBSA} - T\Delta S$.

**Appendix Table 4.** Molecular characterization of GyrA and ParC in *Mycoplasma bovis* isolates that had different susceptibility to enrofloxacin, danofloxacin, and ciprofloxacin*

| | | Amino acid mutations in QRDRs | | Fluoroquinolone MICs, µg/mL | | |
|---|---|---|---|---|---|---|
| *M. bovis* isolates | ST | GyrA | ParC | Enrofloxacin | Danofloxacin | Ciprofloxacin |
| PG45†‡ | 12 | S83 | S80, D84 | 0.125 | 0.125 | 0.25 |
| Madison‡ | 12 | S83 | S80, D84 | 0.125 | 0.125 | 0.25 |
| Z-0093‡ | New | S83 | S80, D84 | 0.125 | 0.125 | 0.25 |
| ZY‡ | New | S83 | S80, D84 | 0.125 | 0.125 | 0.25 |
| GT01‡ | 52 | S83 | S80, D84 | 0.125 | 0.125 | 0.25 |
| WW-1‡ | 52 | S83 | S80, D84 | 0.125 | 0.125 | 0.25 |
| OF2‡ | 52 | S83 | S80, D84 | 0.125 | 0.125 | 0.25 |
| ZY-J4 | 52 | S83F | S80, D84G | 4 | 2 | 4 |
| ZY-J5 | 52 | S83F | S80, D84G | 4 | 2 | 4 |
| ZY-J3 | 52 | S83F | S80R, D84 | 8 | 4 | 8 |
| P-1 | 52 | S83F | S80R, D84 | 8 | 4 | 16 |
| P-2 | 52 | S83F | S80R, D84 | 8 | 4 | 16 |
| NM-1 | 52 | S83Y | S80R, D84 | 8 | 4 | 16 |

|  |  | Amino acid mutations in QRDRs |  | Fluoroquinolone MICs, μg/mL |  |  |
|---|---|---|---|---|---|---|
| *M. bovis* isolates | ST | GyrA | ParC | Enrofloxacin | Danofloxacin | Ciprofloxacin |
| NM-2 | 52 | S83Y | S80R, D84 | 8 | 4 | 16 |
| NM-3 | 52 | S83Y | S80R, D84 | 8 | 4 | 16 |

*QRDR, quinolone resistance-determining regions; ST, sequence type.

†Standard strain of *Mycoplasma bovis*.

‡Clinical isolate with no amino acid mutations in the GyrA or ParC proteins.

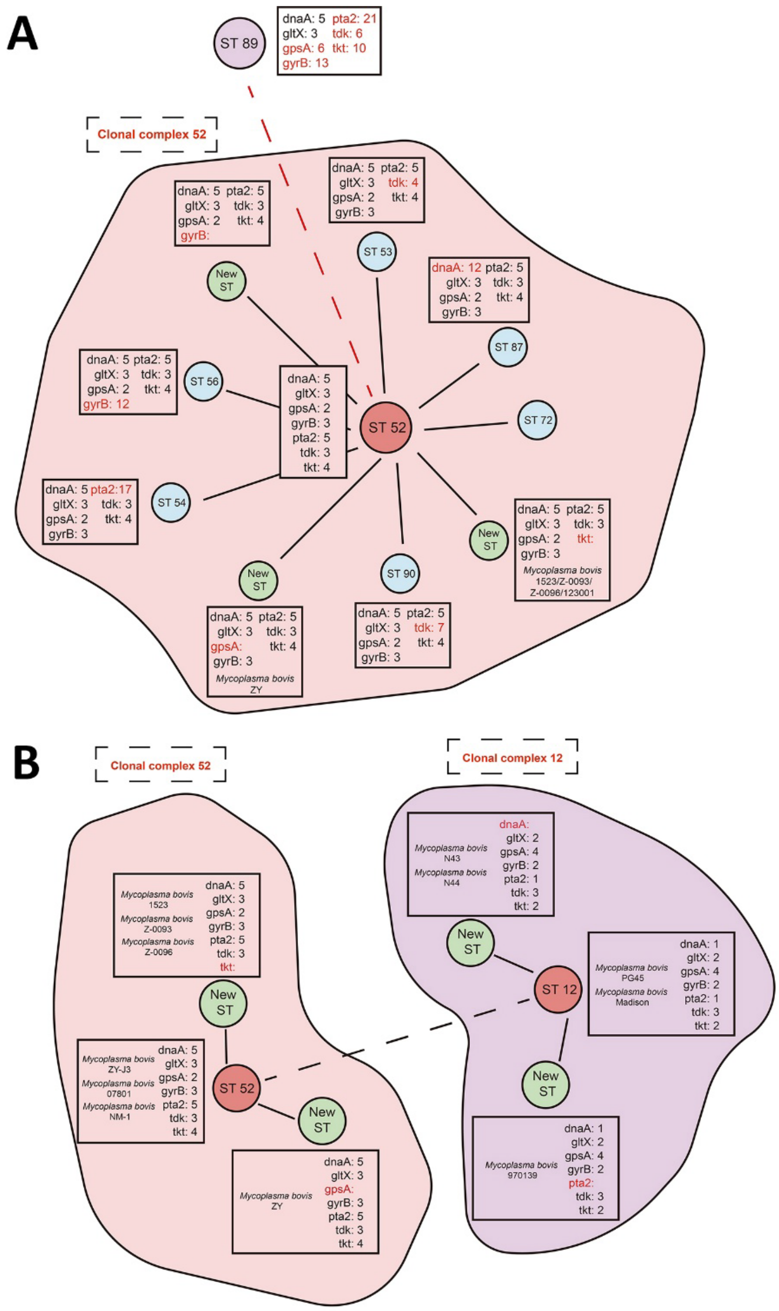


**Appendix Figure 1.** Multilocus sequence typing genotypes of prevalent *M. bovis* isolates in China. A) Clonal complex (CC) 52 is formed with ST52 (red) as the core genotype. Blue indicates other ST genotypes belonging to CC52. Green indicates new ST genotypes belonging to CC52. Purple indicates strains that do not belong to CC52. B) Analysis of *M. bovis* isolates from CC52 and CC12. *M. bovis* strains 1523, Z-0093, Z-0096, and ZY belong to the CC52 with ST52 as the core genotype. *M. bovis* strains N43, N44, and 970139 belong to the CC12 with ST12 as the core genotype. ST, sequence type.

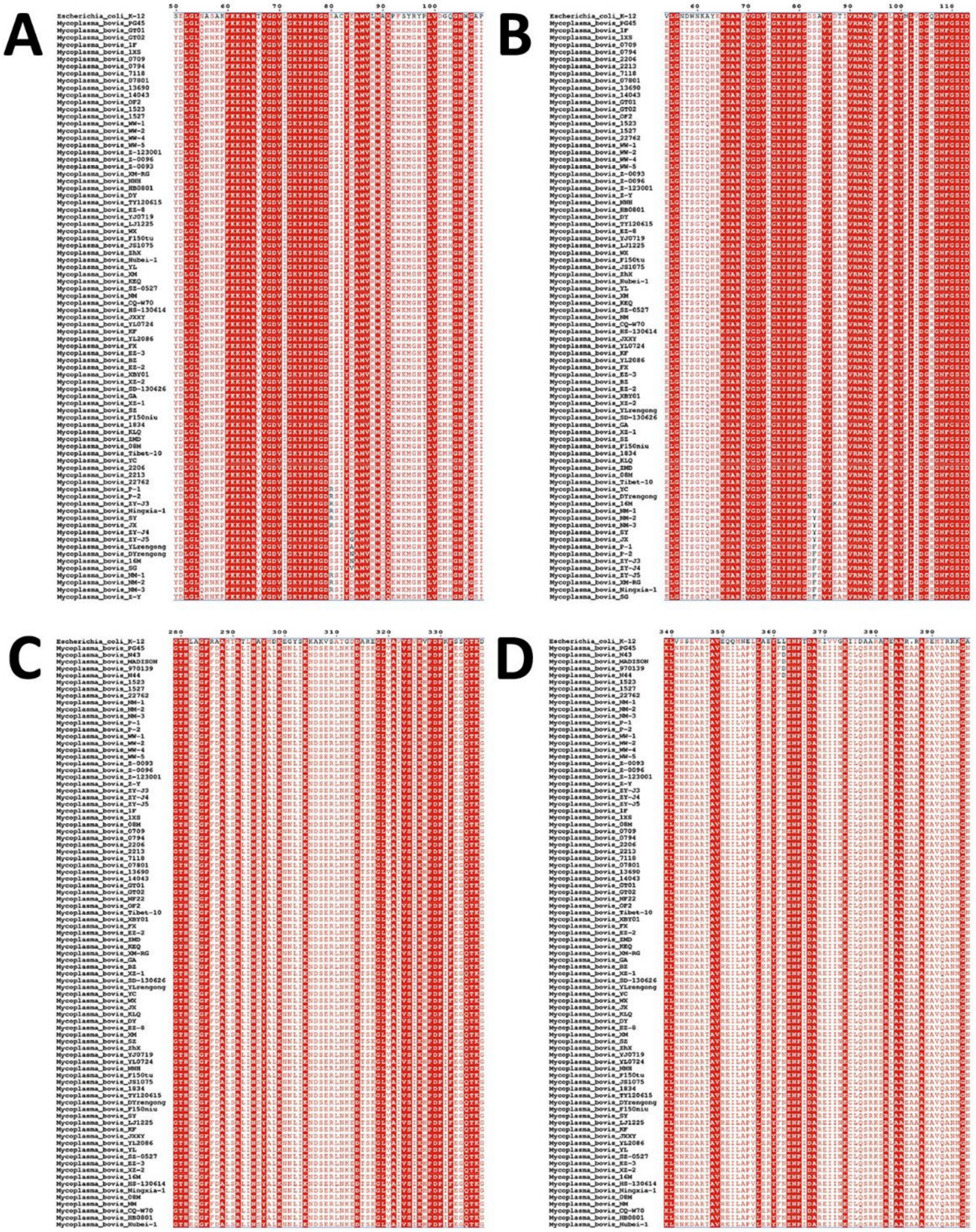

**Appendix Figure 2.** Amino acid sequence alignments of proteins from *Mycoplasma bovis* isolates from China. A, B) Multiple alignments of ParC (A) and GyrA (B) amino acid sequences indicating mutations. C, D) Sequence alignments of key quinolone resistance determining regions within GyrB. Regions surrounding the GyrB Val320 residue (C) and Asp362 residue (D).

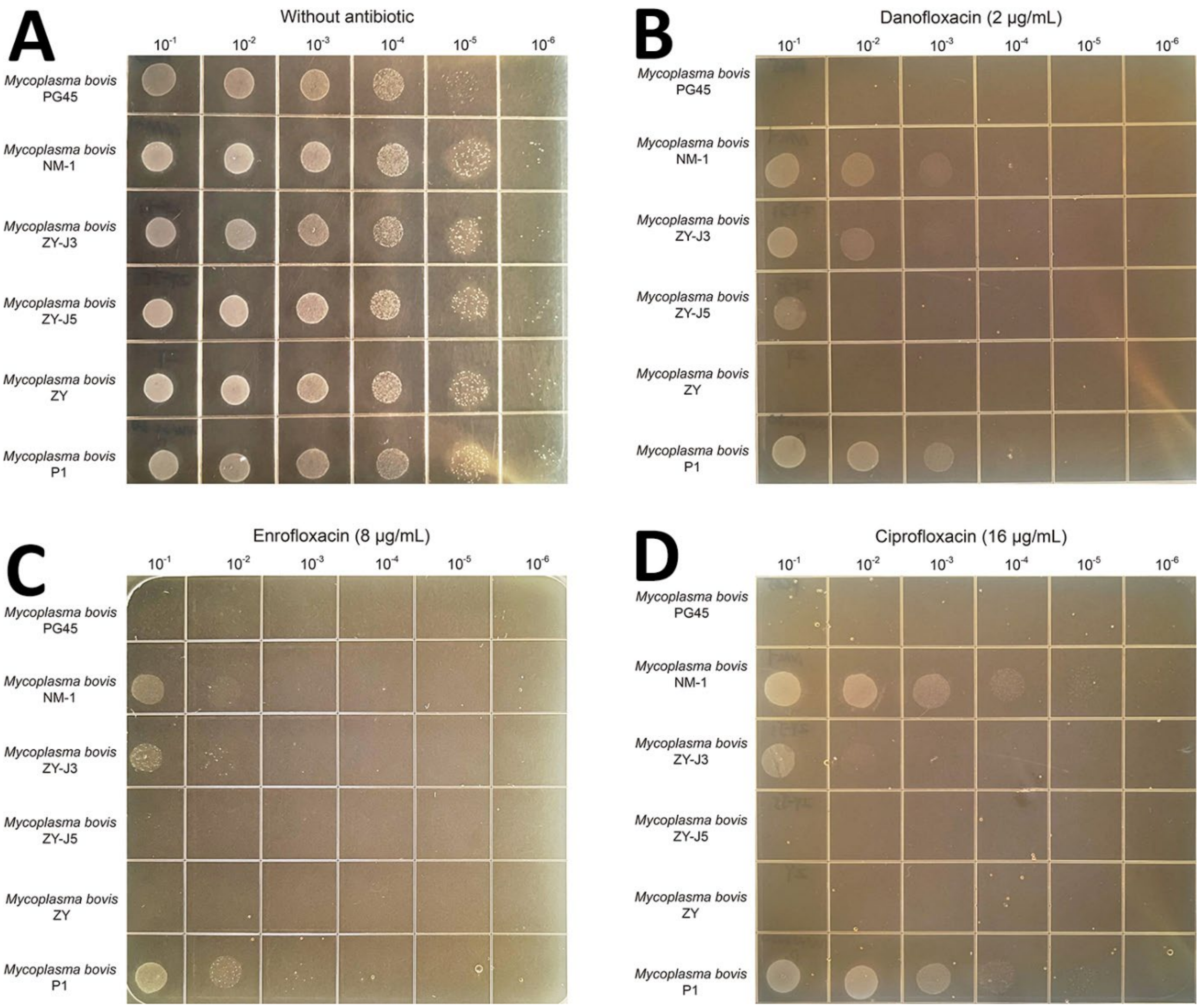


**Appendix Figure 3.** Drug susceptibility spot-plate assay of *Mycoplasma bovis* ParC and GyrA mutant strains. A–D) Spot-plate assay of *M. bovis* isolates on agar medium containing no antibiotic (A), danofloxacin (B), enrofloxacin (C), and ciprofloxacin (D). *M. bovis* nonmutant strains PG45 and ZY were used as controls.

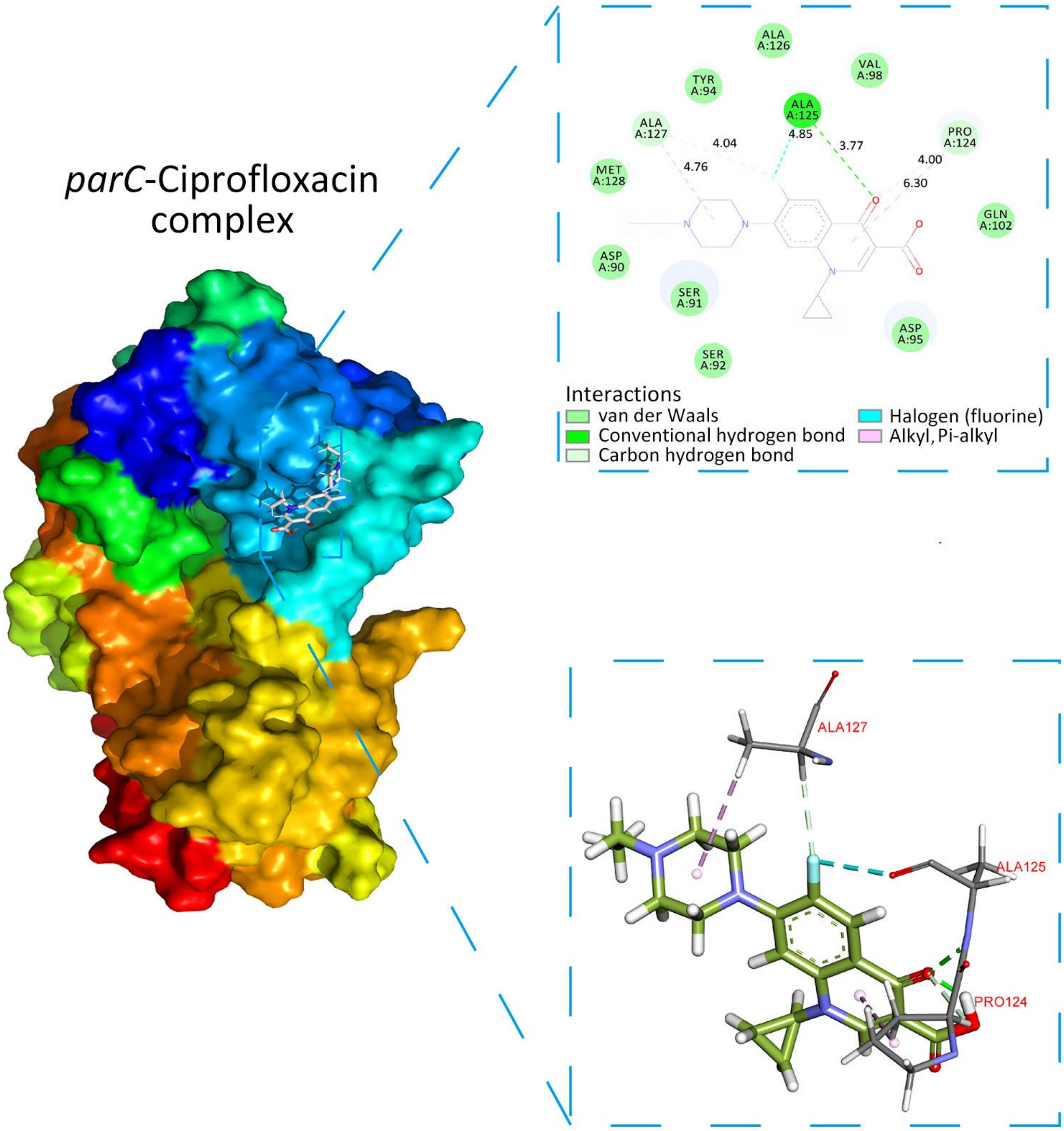


**Appendix Figure 4.** Molecular dynamic simulation of wild-type *Mycoplasma bovis* ParC protein bound to ciprofloxacin. ALA125 in ParC protein forms a hydrogen bond with the small-molecule ligand ciprofloxacin, whereas ALA127 and PRO124 form Pi-alkyl and alkyl hydrophobic interactions, and residues, such as SER91, TYR94, and VAL98, form van der Waals interactions with the small-molecule ligand. Zoomed areas show specific amino acid interactions with the drug.
